# Supplementary material for: Juvenile Hormone as a contributing factor in establishing midgut microbiota for fecundity and fitness enhancement in adult female Aedes aegypti
Source: Commun Biol. 2024 Jun 5;7:687. doi: 10.1038/s42003-024-06334-y (PMC11153597; doi:10.1038/s42003-024-06334-y)
Supplement: Supplementary file 2 — Supplementary Information [file 42003_2024_6334_MOESM2_ESM.pdf]

Supplementary Information: Juvenile Hormone as a Contributing Factor in Establishing Midgut Microbiota for Fecundity and Fitness Enhancement in Adult Female *Aedes aegypti.*

Mabel L. Taracena-Agarwal*[1,2,3,4], Ana Beatriz Walter-Nuno[1,2], Vanessa Bottino-Rojas[1,2], Alessandra Paola Girard Mejia[4], Kelsey Xu[4], Steven Segal[4], Ellen M. Dotson[3], Pedro L. Oliveira[1,2], Gabriela O. Paiva-Silva*[1,2]

Supplementary Table 1. qRT-PCR Primer List.

Gene	VectorBase ID	Primer name	Sequence
Ribosomal Protein 7	AAEL009496	RP7_F	GGGACAAATCGGCCAGGCTATC
		RP7_R	TCGTGGACGCTTCTGCTTGTTG
Actin	AAEL011197	act_F	CCATGTACCCAGGTATTGCT
		act_R	ATCTGTTGGAAGGTGGACAG
Attacin	AAEL003389	att_F	TTGGCAGGCACGGAATGTCTTG
		att_R	TGTTGTCGGGACCGGGAAGTG
Deffensin A	AAEL027792	def_F	GATTCGGCGTTGGTGATAGT
		def_R	TTATTCAATTCCGGCAGACG
Gambicin	AAEL004522	gam_F	GCCAAAACCTGTTCCTCTTG
		gam_R	CGATGTAGCATTCGGTGATG
Caspase 16	AAEL005956	Cas16_F	TCCGCTATCTTCATATTGTATCCTTTG
		Cas16_R	GACCCGCCACTGTATCTCTG
Delta	AAEL025606	del_F	AAGGCAACTGTATCGGAGCG
		del_R	TATGACATCGCCAAACGTGC
Bacterial 16S		B16S_F	TCCTACGGGAGGCAGCAGT
		B16S_R	GGACTACCAGGGTATCTAATCCTGTT

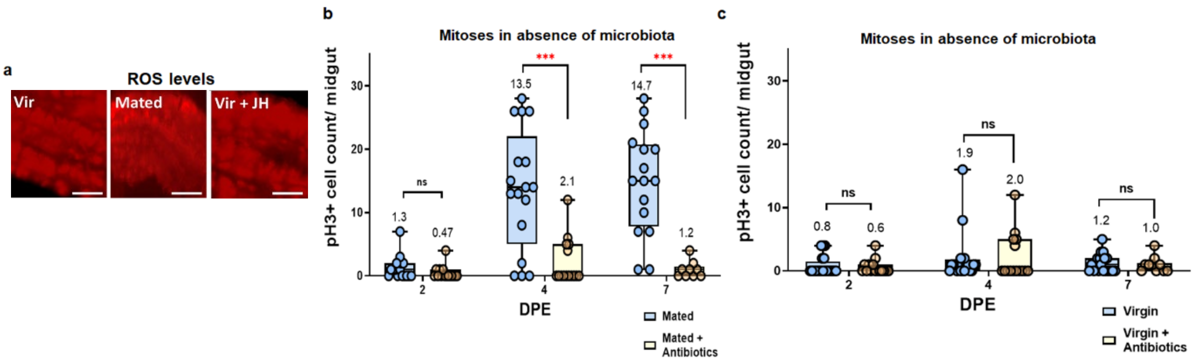

Supplemental Figure 1. ROS levels are independent of mating status and cell proliferation in the midgut of *Ae. aegypti* females is reduced in the absence of normal microbiota. (a) Fluorescence levels in the midgut epithelium of all groups did not reveal any differences between mated, virgin, and virgin treated with JH mosquitoes. DHE measures ROS directly in live cells and therefore ROS presence is directly represented as total DHE fluorescence. Scale bar = 100 µm. For all groups, 2 mM DHE was used and experimental conditions were identical n > 10, three replicates. **(b)** Cell proliferation in the midgut epithelium was significantly lower in the groups of mated females fed with antibiotics and therefore lacking the midgut microbiota. *** P < 0.001 (T-test). **(c)** Cell proliferation in the midgut epithelium did not change in the groups of virgin females fed with antibiotics. ns P > 0.05 (T-test).

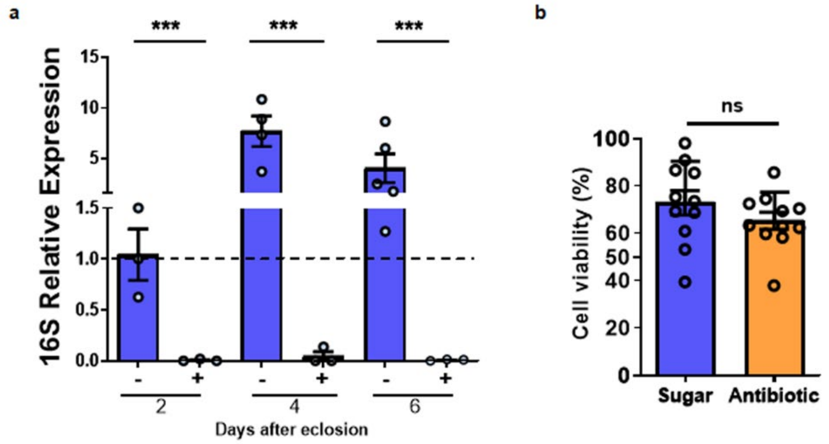

Supplemental Figure 2. Antibiotic treatment impaired midgut microbiota establishment without causing cellular toxicity. (a) Microbiota depletion was confirmed by qRT-PCR in groups treated with antibiotics. Each experiment was performed three times, with three samples of at least ten posterior midguts. *** P < 0.001 (T-test). **(b)** Lack of cell toxicity was confirmed

in the same groups (sugar-fed and sugar-fed with antibiotics) through MTT assays. Each experiment was performed three times.

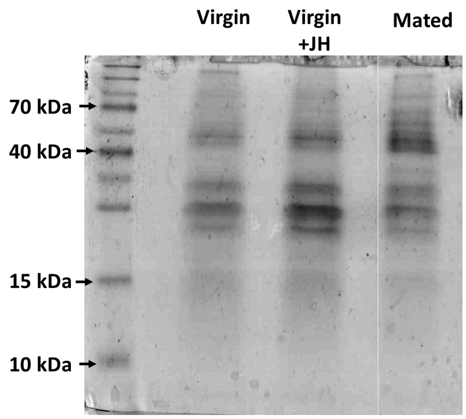

Supplemental Figure 3. Protein profiles from eggs laid by virgin females treated with JH did not show significant differences from the acetone-treated controls. *Ae. aegypti* red eye strain adult females (virgin, virgin with JH, and mated) were blood-fed to study the protein composition of the eggs oviposited by each group. 80 fresh laid eggs were collected and homogenized, and the crude egg extract supernatant was used for protein profile analysis. 15% SDS-PAGE gels with a constant voltage of 90 mV were run, and the gels were stained with Coomassie Blue G and destained with distilled water plus methanol 40%. The protein profile observed in the eggs from virgin females remained unchanged. Eggs from mated females presented a slightly divergent profile.

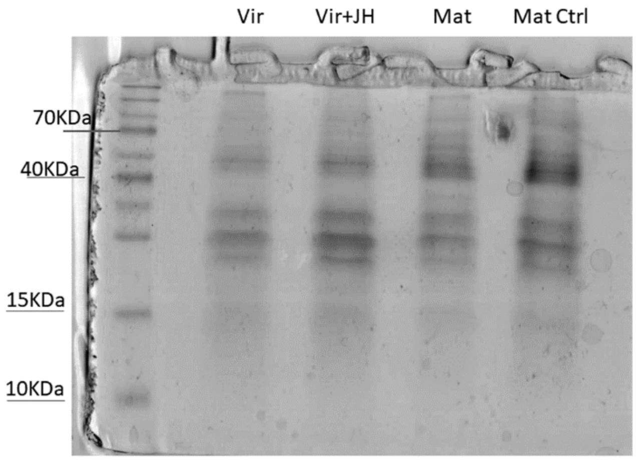

Supplemental Figure 4. Unedited and uncropped picture of the protein gel for Supplemental Figure 3.
