# Supplementary material for: Juvenile Hormone as a contributing factor in establishing midgut microbiota for fecundity and fitness enhancement in adult female Aedes aegypti
Source: Commun Biol. 2024 Jun 5;7:687. doi: 10.1038/s42003-024-06334-y (PMC11153597; doi:10.1038/s42003-024-06334-y)
Supplement: Supplementary file 3 — reporting summary [file 42003_2024_6334_MOESM3_ESM.pdf]

nature portfolio

Corresponding author(s): Mabel L Taracena-Agarwal
Gabriela Oliveira Paiva-Silva

Last updated by author(s): 2024/04/10

Reporting Summary

Nature Portfolio wishes to improve the reproducibility of the work that we publish. This form provides structure for consistency and transparency in reporting. For further information on Nature Portfolio policies, see our Editorial Policies and the Editorial Policy Checklist.

Statistics

For all statistical analyses, confirm that the following items are present in the figure legend, table legend, main text, or Methods section.

n/a	Confirmed	
☐	☒	The exact sample size (*n*) for each experimental group/condition, given as a discrete number and unit of measurement
☐	☒	A statement on whether measurements were taken from distinct samples or whether the same sample was measured repeatedly
☐	☒	The statistical test(s) used AND whether they are one- or two-sided<br>*Only common tests should be described solely by name; describe more complex techniques in the Methods section.*
☐	☒	A description of all covariates tested
☐	☒	A description of any assumptions or corrections, such as tests of normality and adjustment for multiple comparisons
☐	☒	A full description of the statistical parameters including central tendency (e.g. means) or other basic estimates (e.g. regression coefficient) AND variation (e.g. standard deviation) or associated estimates of uncertainty (e.g. confidence intervals)
☐	☒	For null hypothesis testing, the test statistic (e.g. *F*, *t*, *r*) with confidence intervals, effect sizes, degrees of freedom and *P* value noted<br>*Give P values as exact values whenever suitable.*
☒	☐	For Bayesian analysis, information on the choice of priors and Markov chain Monte Carlo settings
☒	☐	For hierarchical and complex designs, identification of the appropriate level for tests and full reporting of outcomes
☒	☐	Estimates of effect sizes (e.g. Cohen's *d*, Pearson's *r*), indicating how they were calculated

Our web collection on statistics for biologists contains articles on many of the points above.

Software and code

Policy information about availability of computer code

Data collection	no software was used.
Data analysis	Results were analyzed using GraphPad Prism 10.2

For manuscripts utilizing custom algorithms or software that are central to the research but not yet described in published literature, software must be made available to editors and reviewers. We strongly encourage code deposition in a community repository (e.g. GitHub). See the Nature Portfolio guidelines for submitting code & software for further information.

Data

Policy information about availability of data

All manuscripts must include a data availability statement. This statement should provide the following information, where applicable:

- Accession codes, unique identifiers, or web links for publicly available datasets
- A description of any restrictions on data availability
- For clinical datasets or third party data, please ensure that the statement adheres to our policy

The data that support the findings of this study are available from the corresponding author, M.L.T. and G.O.P.S., upon reasonable request.

Research involving human participants, their data, or biological material

Policy information about studies with human participants or human data. See also policy information about sex, gender (identity/presentation), and sexual orientation and race, ethnicity and racism.

Reporting on sex and gender	No human subjects were used in this study.
Reporting on race, ethnicity, or other socially relevant groupings	No human subjects were used in this study.
Population characteristics	No human subjects were used in this study.
Recruitment	No human subjects were used in this study.
Ethics oversight	No human subjects were used in this study.

Note that full information on the approval of the study protocol must also be provided in the manuscript.

Field-specific reporting

Please select the one below that is the best fit for your research. If you are not sure, read the appropriate sections before making your selection.

☒ Life sciences ☐ Behavioural & social sciences ☐ Ecological, evolutionary & environmental sciences

For a reference copy of the document with all sections, see nature.com/documents/nr-reporting-summary-flat.pdf

Life sciences study design

All studies must disclose on these points even when the disclosure is negative.

Sample size	Sample sizes were chosen on the basis of precedent in the literature or general statistical guidelines that included a minimum of three independent biological replicates.
Data exclusions	No data were excluded from the results reported.
Replication	All reported results were replicated using independently generated biological samples.
Randomization	Individuals that were analyzed for a given treatment were randomly selected from the rearing containers holding Aedes aegypti mosquitoes of a specific age.
Blinding	Blinding was not possible given the conditions of the laboratories and the protocols followed by the authors.

Reporting for specific materials, systems and methods

We require information from authors about some types of materials, experimental systems and methods used in many studies. Here, indicate whether each material, system or method listed is relevant to your study. If you are not sure if a list item applies to your research, read the appropriate section before selecting a response.

Materials & experimental systems

n/a	Involved in the study	
☒	☐	Antibodies
☒	☐	Eukaryotic cell lines
☒	☐	Palaeontology and archaeology
☐	☒	Animals and other organisms
☒	☐	Clinical data
☒	☐	Dual use research of concern
☒	☐	Plants

Methods

n/a	Involved in the study	
☒	☐	ChIP-seq
☒	☐	Flow cytometry
☒	☐	MRI-based neuroimaging

Animals and other research organisms

Policy information about studies involving animals; ARRIVE guidelines recommended for reporting animal research, and Sex and Gender in Research

Laboratory animals	No vertebrate animals were used in the study. only insects were used. Aedes aegypti Red eyeand Rockefeller strains.
Wild animals	No wild animals were used in this study.
Reporting on sex	All findings concern to female mosquitoes, as it is indicated throughout the text, legends and methods.
Field-collected samples	No field collected samples were used in this study.
Ethics oversight	At the Federal University of Rio de Janeiro (UFRJ), all animal care and experimental protocols were conducted in accordance with the guidelines of the Committee for Evaluation of Animal Use for Research (CAUAP-UFRJ). The protocols were approved by CAUAP-UFRJ in 2013 under registration number IBQM155/13. At the Centers for Disease Control and Prevention (CDC), laboratory stocks and rearing protocols for the Ae. aegypti used were obtained from the Biodefense and Emerging Infections Resources/ Malaria Research and Reference Reagent Resource Center (BEI/MR4).

Note that full information on the approval of the study protocol must also be provided in the manuscript.

Plants

Seed stocks	No plants were used in this study.
Novel plant genotypes	n/a
Authentication	n/a
